# Cumulative incidence of type 1 diabetes in two cohorts of children with different national gluten recommendations in infancy

**DOI:** 10.1007/s00592-023-02168-y

**Published:** 2023-08-17

**Authors:** Marie Lindgren, Elsa Palmkvist, Fredrik Norström, Mara Cerqueiro Bybrant, Anna Myleus, Ulf Samuelsson, Johnny Ludvigsson, Annelie Carlsson

**Affiliations:** 1 Department of Clinical Science, Lund University, Lund, Sweden; 2 Children’s Clinic, Vrinnevi Hospital, Norrköping, Sweden; 3 Department of Epidemiology and Global Health, Umeå University, Umeå, Sweden; 4 Paediatric Endocrinology Unit, Department of Women’s and Children’s Health, Karolinska Institutet, Stockholm, Sweden; 5 Department of Public Health and Clinical Medicine, Family Medicine, Umeå University, Umeå, Sweden; 6 Crown Princess Victoria’s Children’s Hospital, Region Östergötland, Linköping, Sweden; 7 Division of Paediatrics, Department of Biomedical and Clinical Sciences, Linköping University, Linköping, Sweden; 8 Department of Pediatric, Skånes University hospital, Lund, Sweden

**Keywords:** Paediatric type 1 diabetes, Infant feeding, Gluten, Early childhood risk factors, Celiac disease

## Abstract

**Aims:**

Between 1985 and 1996, Sweden experienced an “epidemic” of celiac disease with a fourfold increase in incidence in young children. Timing and amount of gluten introduced during infancy have been thought to explain this “epidemic”. We aimed to study whether the cumulative incidence of type 1 diabetes differs between children born during the “epidemic” compared to children born after.

**Methods:**

This is a national register study in Sweden comparing the cumulative incidence of type 1 diabetes in two birth cohorts of 240 844 children 0–17 years old born 1992–1993, during the “epidemic”, and 179 530 children born 1997–1998, after the “epidemic”. Children diagnosed with type 1 diabetes were identified using three national registers.

**Results:**

The cumulative incidence of type 1 diabetes by the age of 17 was statistically significantly higher in those born after the “epidemic” 0.77% than in those born during the “epidemic” 0.68% (*p* < 0.001).

**Conclusion:**

The incidence of type 1 diabetes is higher in those born after the epidemic compared to those born during the epidemic, which does not support the hypothesis that gluten introduction increases the incidence of T1D. Changes in gluten introduction did not halt the increased incidence of type 1 diabetes in Sweden.

## Introduction

The incidence of type 1 diabetes (T1D) in Sweden is, next to Finland, the highest in the world [1]. During two decades, from early 1980s to the beginning of 2000, Sweden [2, 3] and many other countries [1, 4, 5] recognised an increase in incidence of children diagnosed with T1D, in Europe the incidence rate rose by approximately 3–4% yearly [6].

The vast majority of children with T1D have the human leukocyte antigen (HLA) DQ2 and/or DQ8 within the HLA area on chromosome 6 [7], but the fast increase in T1D incidence during the last decades cannot be explained by changes in the genetic upset in the population, rather environmental factors must play a major role in the aetiology of the disease [8, 9]. Environmental factors such as infections, diet, toxins, increased body mass index (BMI) and increased sedentary lifestyle in childhood have been proposed to contribute to T1D in children [9, 10].

Celiac disease (CD) shares the genetic risk alleles with T1D and gluten is the known trigger for CD [11]. Celiac disease and T1D have a well-known co-morbidity [12] and gluten has been discussed as one of the factors in our diet that can be involved in the pathogenesis of T1D. One hypothesis is that gluten may affect the leakiness of the intestinal mucosa, the gut microbiota and/or the immune system leading to islet autoimmunity (IA) [13, 14]. A gluten-free diet has in some small studies been shown to have a beneficial effect on the preservation of beta cell function and thereby improve insulin secretion in humans [14]. In children, both the amount of ingested gluten and the timing and mode of gluten introduction have been studied as having diabetogenic potential, but the results are inconsistent [15–24]. For CD most studies show that timing of gluten introduction in infancy does not affect the risk of later CD diagnosis but data on amount of gluten are inconclusive [25]

*The Swedish epidemic of celiac disease* is a concept referring to a period of time with a dramatically higher incidence of CD in young Swedish children below the age of two years [26]. The “epidemic” of CD was observed in children born between 1985 and 1996 [26]. Concomitantly, there was a national change in Swedish infant feeding recommendations to postpone the gluten introduction in infancy from 4 to 6 months, which practically was from a gradual introduction of gluten at four-month age before 1982, to a more abrupt gluten introduction at 6 months of age. In addition, the gluten content in the cereal based follow-on formula was concurrently increased. In 1996 the feeding recommendations were changed back to introduction at 4 months of age, and the gluten content in formulas was simultaneously decreased again [26]. After these changes, a rapid decrease in CD incidence in young children started 1995, approaching levels in incidence rate as before the “epidemic” [27, 28].

As a result, Sweden has a unique situation with birth cohorts that differ with respect to recommended gluten introduction and presumed different amounts of gluten intake in infancy. It was shown in the Swedish study ETICS (exploring the iceberg of celiac disease in Sweden), in which almost a tenth of the children born 1993 and 1997 were screened at 12 years of age for CD, that children born 1993, during the “epidemic”, had a statistically significantly higher prevalence of CD also at 12 years of age, compared to 12-year-old children born in 1997 just after the “epidemic” of CD. The abrupt gluten introduction and the increased gluten content in the food in infancy was believed to play an important role in this difference in CD prevalence’s [28].

Our aim was to study whether children born during and after the “epidemic” of CD also differ in the risk of T1D. A previous study reported an increasing incidence of T1D in children in Sweden, and also showed that the increased incidence tapered after the year 2000 [2]. In that study, children born during the epidemic were mixed with children born after the epidemic. In this study we focused on two unique birth cohorts with short time difference between the cohorts and also known CD prevalence. Our hypothesis was that the infant feeding practices and the increase amount of gluten during the “epidemic” did not only increase the risk of CD, but also increased the risk of developing T1D in children born during the “epidemic” of CD compared to children born after the “epidemic”.

## Methods

This is a population-based study using Swedish national registers to compare the cumulative incidence of T1D in children 0–17 years old in two cohorts. The “epidemic cohort” born 1992–1993, during the “epidemic” of CD, comprised of 240 844 children and the “post-epidemic cohort” born 1997–1998, after the “epidemic” of CD, comprised of 179 530 children [29].

These two cohorts were chosen because we wanted to have two cohorts with a short timespan to avoid as many unknown confounders as possible. For the birth cohorts 1993 and 1997, we know the true prevalence of CD [28] and to gain power we added 1992 and 1998.

Data regarding T1D diagnosis, birth year and age/date at T1D diagnosis in the two cohorts was collected from The Swedish National Inpatient Register (IPR), SWEDIABKIDS and the Swedish National Diabetes Register (NDR). IPR is administered by the Swedish National Board of Health and Welfare [30], in the register T1D is defined by the following codes from the International Classification of Disease (ICD) 9^th^ and 10^th^ editions: 250A-X (ICD9) or E10.0–9 (ICD10). SWEDIABKIDS is an ongoing national longitudinal quality register for children up to 18 years old [31]. The Swedish National Diabetes Register (NDR) is an ongoing longitudinal register for adults with diabetes [32].

Data was collected from the registers from 1st of January 1992 to 31st of December 2015.

Included in the study were children born 1992–1993 or 1997–1998 with a T1D diagnosis up to the age of 17 years in either of the registers. Children excluded from the study were those;

- T1D diagnosis in Swediabkids/NDR but not in IPR.
- Children diagnosed with T1D in IPR but another diabetes type than T1D in Swediabkids (type 2 diabetes, Maturity-onset diabetes in the youth, secondary diabetes or another type of diabetes)
- Children with T1D diagnosis only in the part of the IPR that registers hospital-based outpatient care, since children with T1D very seldom get their diagnosis at an outpatient visit.

Data of population, immigration and mortality, with gender specified, were collected from Statistics Sweden [29]. In children 17 years old, 9% were born outside Sweden in 2010 (born 1993) and 16% in 2015 (born 1998), corresponding to 7 000 more immigrants in cohort 1997–1998 compared to cohort 1992–1993.

The study was approved by the Research Ethics Committee Lund (Dnr 2014/476 EPN).

### Statistical methods and data analysis

Statistical analyses were conducted in IBM SPSS Statistics Version 25 and in excel 2016. The cumulative incidence of T1D and comparisons of continuous data were compared with independent *t*-test. A *p* < 0.05 was considered to be statistically significant.

## Results

**Fig. 1:**
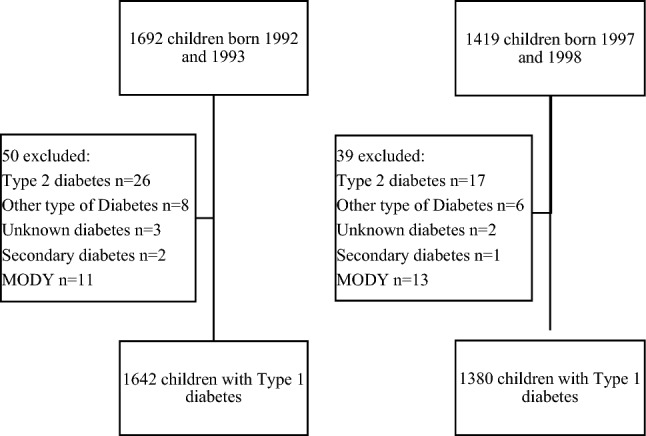
Children excluded in the cohorts

In total, there were 3111 children with a diabetes diagnosis up to the age of 17.0 years: 1692 born in the epidemic cohort 1992–1993 and 1429 born in the post-epidemic cohort 1997–1998. In the epidemic cohort, 50 children were excluded leaving 1642 children (732 girls), while in the post-epidemic cohort 39 children were excluded leaving 1380 children (628 girls), Fig. 1.

The proportion of girls was similar in both cohorts, 48.7% in those born 1992–1993 and 48.5% in those born 1997–1998. In Table 1, the demographic characteristics of the cohorts are presented.

**Table 1:** Demographic characteristics of the epidemic cohort born 1992–1993 during the “Swedish celiac disease (CD) epidemic” and the post-epidemic cohort born 1997–1998 after the “Swedish CD epidemic”

	Epidemic cohort	Post-epidemic cohort
*Birth cohort*	240 844	179,530
*Sex (%)*		
Girls	117 323 (48.7)	87 079 (48.5)
Boys	123 521 (51.3)	92 451 (51.5)
*Type 1 diabetes diagnosis (%)*		
Birth cohort	1642	1380
Girls	732 (44.6)	628 (45.5)
Boys	910 (55.4)	752 (54.5)
*Mean age at type 1 diabetes diagnosis (SD)*		
Birth cohort	9.8 years (4.2)	9.5 years (4.2)
Girls	9.3 years (3.9)	9.1 years (4.0)
Boys	10.2 years (4.3)	9.9 years (4.3)

### Cumulative incidence of type 1 diabetes

**Fig. 2:**
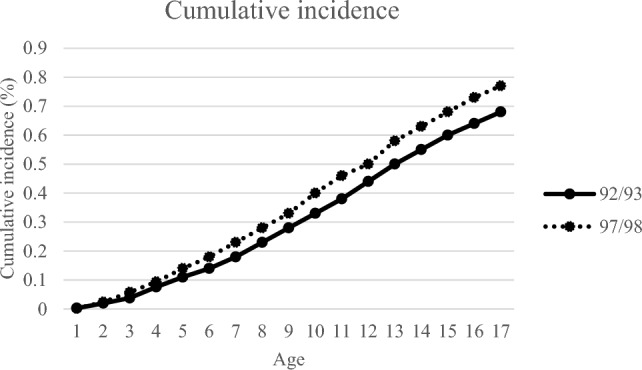
Cumulative incidence of type 1 diabetes in children followed from birth to 17 years’ birthday in the epidemic cohort born 1992/1993 and the post-epidemic cohort born 1997/1998

The cumulative incidence of T1D in the epidemic cohort was 0.68% and in the post-epidemic cohort 0.77% at the age of 17.0, Fig. 2. The cumulative incidence of T1Ds was statistically significantly higher in the post-epidemic cohort than in the epidemic cohort, *p* < 0.001.

The cumulative incidence of T1D was statistically significantly higher for both girls (0.62% vs 0.72%) and boys (0.74% vs 0.81%), respectively, in those born 1997–1998 than in those born 1992–1993 (*p* = 0.007 in girls and *p* < 0.001 in boys).

### Age at type 1 diabetes diagnosis

Mean age at T1D diagnosis was 9.8 years (median 10.2 years) in the epidemic cohort and 9.5 years (median 9.8 years) in the post-epidemic cohort 1997–1998. Mean age at T1D diagnosis was statistically significantly lower in girls compared to boys both in the 1992–1993 cohort *p* < 0.001 and in the 1997–1998 cohort *p* < 0.001, (Table 1), but there was no difference in mean age at T1D diagnosis between girls in the epidemic vs post-epidemic cohort, nor in boys in the epidemic vs post-epidemic cohort.

The incidence of T1D in children 0–17 years was statistically significantly higher in the post-epidemic cohort in those 2–10 years of age (Table 2).

**Table 2:** The incidence of type 1 diabetes per 100,000 children 0–17 years, in the epidemic cohort born 1992–1993 during the “Swedish celiac disease (CD) epidemic” and the post-epidemic cohort born 1997–1998 after the “Swedish CD epidemic”, based on age at type 1 diabetes diagnosis

Age groups (years)	Epidemic cohort	Post-epidemic cohort	*P*-value (95% confidence interval)
0–1.99	22.42	27.85	0.268 (− 4.2–15.0)
2–4.99	89.27	113.63	0.013 (5.1–43.6)
5–9.99	222.97	257.9	0.021 (5.2–64.7)
10–14.99	271.13	288.53	0.29 (− 14.8–49.6)
15–17	75.98	80.76	0.583 (− 12.3–21.8)

## Discussion

In these unique birth cohorts, we have shown that the cumulative incidence of T1D in children followed from birth to the age of 17, was higher in the birth cohort born after the Swedish “epidemic” of CD, than in the birth cohort born during the Swedish “epidemic” of CD. The higher cumulative incidence of T1D after the “epidemic” of CD was seen in both boys and girls.

The result with a higher cumulative incidence of T1D in the post-epidemic birth cohort, also when we dissect the cohorts into birth cohorts born during and after the “epidemic” of CD, is in line with the study by Berhan et al. [2], but contradicts our hypothesis; that the infant feeding practices during the “epidemic” of CD, with a later and more abrupt introduction of gluten in larger amounts could lead to an increased risk of T1D in parallel with the increased prevalence of CD [28]. On the contrary, we found a higher risk of T1D after the “epidemic” of CD when parents were recommended to introduce gluten slowly, in small amounts, at 4 months of age.

If gluten has anything to do with the difference in cumulative incidence seen between the two cohorts, a gradual and early introduction of gluten seems to trigger the development of T1D and a late introduction seems to be protective according to our results. Early gluten introduction has been shown, in a small number of study participants in other studies, to increase the risk of islet autoimmunity (IA) [15, 16] and T1D [16, 21, 22] in children at high-risk for T1D, but in those studies gluten introduction was already before 3–4 months of age.

A late gluten introduction, after 6 months of age, has been compared with gluten introduction at 4–6 month of age, but also with inconsistent results. Most studies have not shown any differences in risk [16, 18–20, 22], but some report increased risk if gluten is introduced in the food after 7 months of age [15]. In the TEDDY-study (investigating children with a genetic risk for T1D), children with gluten introduction after 9 months of age have a higher risk of IA than those introduced to gluten at 4–9 months of age [17]. A gluten-free diet has in small human studies been shown to have a potential to reduce the risk of T1D [14, 33] and also when introduced after the diagnosis of T1D to prolong the remission [34].

In our study, not only the timing of gluten introduction differed between the cohorts but also the amount of gluten, where the epidemic cohort was introduced to gluten in larger amounts [28]. The amount of gluten intake has been studied in Norway, where it was shown that the amount of gluten at 18 month of age was associated with an increased risk of T1D during follow-up [24]. Also in Finland a higher amount of gluten-containing food up to six years of age was associated with an increased risk of IA [23]. In contrast, the DAISY study, which consisted of children with a high genetic risk for T1D, concluded that there was no association between gluten intake at 1–2 years of age or throughout childhood and the risk of IA and T1D [22].

Taken together, the results from our study and others are inconsistent concerning gluten as a trigger for T1D. From our results, we cannot tell whether it is the change in gluten introduction or something else in the environment that explain the rise in cumulative incidence in the cohort born after the “epidemic” of CD. However, as mentioned above the incidence of T1D has increased during the last decades in Sweden [2, 3], but this trend has been seen in most western countries [1, 4, 5] without epidemic-like incidence patterns in CD as in Sweden. Therefore, it seems unlikely that infant feeding practise with respect to gluten is the explanation for the higher risk of T1D in the post-epidemic cohort compared to the epidemic cohort. Also before as well as after the Swedish “epidemic” of CD, the incidence of T1D increased and it is still increasing, at least in Sweden [35]. So there are other environmental triggers causing this increase.

In accordance with the accelerator hypothesis [10], some studies suggest that a contributing factor may be the rising prevalence in obesity among children [36–39]. According to the accelerator hypothesis growth and excessive weight gain is associated with an increased insulin demand and insulin resistance that accelerate beta-cell apoptosis and autoimmunity in the presence of the susceptibility HLA genotypes leading to T1D [10]. A newly published study present findings that support a link between higher childhood body size and the risk of being diagnosed with T1D [40]. A Swedish study, including children from the ETICS, showed that 12-year-old children born 1997, after the “epidemic” of CD, had a higher BMI compared to children born in 1993, during the “epidemic” of CD, and the prevalence of overweight was also 2% higher for the children born in 1997 compared to the ones born four years earlier [41]. This change in BMI, with increasing insulin requirement, might contribute to our result with an increase in incidence of T1D in the cohort born after the “epidemic” of CD.

Is the difference in cumulative incidence between the birth cohorts of clinical importance? A cumulative incidence of 0.68% vs 0.77% seems almost the same but expressed in individuals per 100,000 with T1D it means that in the post-epidemic cohort 90 per 100,000 more children between 0–17 years old have been diagnosed with T1D compared with the incidence during the epidemic 1992–1993.

The strength of this study is that Sweden has a unique situation with birth cohorts having different infant feeding patterns shown by the “epidemic” of CD. We were also able to analyse two nationwide population-based birth cohorts with only 4–6 years apart, limiting other changes in the society to affect our result. Most other studies of gluten and its role for the development of T1D has been in genetically high-risk children defined as either having high risk HLA and/or having first degree relatives with T1D and most have the appearance of IA as ending point. IA mark the destructive process that can be present for years prior to the diagnosis of T1D, but not always progress to the disease [8]. In this study, we have studied the general population with T1D as end-point.

By using more than one register to define children with T1D we believe that we have an accurate inclusion of children with T1D born during the studied years in Sweden since both SWEDIABKIDS and IPR have high reporting degrees [30, 31].

A limitation is that we do not have individual infant feeding data and cannot make certain that all children born during the “epidemic” of CD did follow the national recommendations on how to introduce gluten in infancy. However we know from the ETICS study that the cohort of children born 1993 had a larger mean intake of gluten from formulas than children born 1997 and it is estimated that 60–70% of the parents did follow the recommendations [28, 42]. Furthermore, we know that this cohort had a higher risk of CD [28], and the abrupt introduction of gluten and/or a larger amounts of gluten, followed by the change in infant feeding recommendations, was believed to play a role in the Swedish “epidemic” of CD.

The birth cohorts were unequal in size, 1992–1993 is larger than the cohort 1997–1998. However, this fits with the fluctuation of number of young parents and newborn babies in Sweden since the increase of babies in the 1940s.

Another limitation is that we have not taken the immigration in to account. There were more children born abroad in the 1997–1998 cohort compared to the 1992–1993 cohort. We did not have any exact numbers on non-Swedish-born children in these cohorts. We do not believe that immigration has affected our results because we know from another study that only 1.5% of children with T1D are born outside of Sweden [43]. We also ignored mortality rate. The mortality of children below 17 years of age is extremely low in both birth cohorts and was regarded as negligible. Another potential loss of follow-up is children moving away from Sweden and diagnosed with T1D in another country, also here we do not believe that this has affected our results.

## Conclusion

The cumulative incidence of T1D in children is higher in a birth cohort born after the Swedish “epidemic” of CD than in a birth cohort born during the Swedish “epidemic” of CD. Different gluten recommendations in infancy did not affect the cumulative incidence of T1D in the same way as it did with CD, nor did it alter the ongoing increase of incidence of T1D in Sweden, a development shared with other European countries.

## Data Availability

The dataset generated and analysed during the current study are not publicly available because the Swedish Data Protection Act (1998:204) does not permit sensitive data from humans (like in our study) to be freely shared. The datasets are available based on ethical permission from the Ethical board in Sweden, from corresponding author (Marie Lindgren).

## Abbreviations

BMI: Body mass index
CD: Celiac disease
ETICS: Exploring the iceberg of celiac disease in Sweden
HLA: Human leukocyte antigen
IA: Islet autoimmunity
IPR: Swedish National Inpatient Register
NDR: The Swedish National Diabetes Register
T1D: Type 1 diabetes
